# Supplementary material for: Content-rich biological network constructed by mining PubMed abstracts
Source: BMC Bioinformatics. 2004 Oct 8;5:147. doi: 10.1186/1471-2105-5-147 (PMC528731; doi:10.1186/1471-2105-5-147)
Supplement: Additional File 2 — The original results of the above study (non-essential files are deleted to keep the file size under the limit set by BMC bioinformatics). [file 1471-2105-5-147-S2.bz2 › chilibotAdditionalFile2/dip50/89ID7962001E326/html/RECO_RECR.html]

 


 **RECO** and **RECR** 
  
Found 42 abstracts in PubMed, retrieved 42.  
 

 What does Google say? 
 PDF only 
| .edu only 

---

**Interactive relationship** (e.g. stimulation, inhibition, etc)

**Neutral relationship**- First,  **RecO**  and  **RecR**  bind to SSB.ssDNA complexes and modify their structure to allow RecA to nucleate on them efficiently.  Ref: 7962001 J Biol Chem, 1994
- ii  **RecO**  and  **RecR**  do not remove SSB from SSB.ssDNA complexes, but instead bind to these complexes.  Ref: 7962001 J Biol Chem, 1994
- i  **RecO**  physically interacts with both  **RecR**  and SSB.  Ref: 7962001 J Biol Chem, 1994
- The interaction between  **RecO**  and SSB is stronger than the  **RecO**   **RecR**  interaction.  Ref: 7962001 J Biol Chem, 1994
- These data suggested that  **RecO**  mediates the interactions of RecF protein with  **RecR**  and with Ssb proteins.  Ref: 8962075 Proc Natl Acad Sci U S A, 1996
- These data suggest that one role of RecF is to stabilize the interaction of  **RecR**  with  **RecO**  in the presence of Ssb protein.  Ref: 8962075 Proc Natl Acad Sci U S A, 1996
- Incubation of RecF,  **RecO** ,  **RecR** , and Ssb proteins resulted in the formation of RecF  **RecO**  Ssb complexes ; i.e.,  **RecR**  was excluded.  Ref: 8962075 Proc Natl Acad Sci U S A, 1996
- Interactions of RecF protein with  **RecO** ,  **RecR** , and single stranded DNA binding proteins reveal roles for the RecF  **RecO**   **RecR**  complex in DNA repair and recombination.  Ref: 8962075 Proc Natl Acad Sci U S A, 1996
- Biochemical interaction of the Escherichia coli RecF,  **RecO** , and  **RecR**  proteins with RecA protein and single stranded DNA binding protein.  Ref: 8483906 Proc Natl Acad Sci U S A, 1993
- We therefore assigned  **recO**  to epistatic group alpha and predict that the  **RecO**  protein acts at the same stage of recombination as the RecF, RecL and  **RecR**  proteins, in a RecFLOR complex.  Ref: 10323239 Mol Gen Genet, 1999
- Preincubation of RecF,  **RecO** , and  **RecR**  proteins prior to addition of Ssb protein resulted in the formation of complexes consisting of RecF,  **RecO** ,  **RecR** , and Ssb proteins.  Ref: 8962075 Proc Natl Acad Sci U S A, 1996
- It is inferred from these results that recF overexpression may reduce UV inducible sulAp expression by a mechanism that is sensitive to the ability of RecF to bind ATP and to the levels of  **RecO**  and  **RecR**  RecOR in the cell, but not to the type of DNA damage per se.  Ref: 7830722 Mol Gen Genet, 1994
- Interactions involving  **RecO**  and  **RecR**  overcome the inhibition of RecA by single stranded DNA binding protein.  Ref: 7962001 J Biol Chem, 1994
- Expression of the chromosomal  **recO**  gene is essential for the  **recR**  suppression.  Ref: 8206844 J Bacteriol, 1994
- On the other hand, the presence of recA2020, a suppressor mutation for recF,  **recO** , and  **recR**  mutations, restored normal UVM in recF,  **recO** , and  **recR**  mutants.  Ref: 9537373 J Bacteriol, 1998

**Non-interactive relationship** (e.g. studied together, co-existance, homology, etc.)

- These results indicate an involvement of the recF,  **recO** , and  **recR**  genes and their products in UVM, possibly by affecting the third role of RecA in UVM.  Ref: 9537373 J Bacteriol, 1998
- RecF,  **RecO** , and  **RecR**  proteins had no effect on the conversion of joint molecules to final products and only appeared to stimulate an early step in the pairing reaction.  Ref: 8483906 Proc Natl Acad Sci U S A, 1993
- RecF protein competes with  **RecO**  protein for  **RecR**  protein association and its addition destabilizes RecAOR filaments.  Ref: 11743007 EMBO J, 2001
- We analyzed effects of overexpression of RecE and RecT on illegitimate recombination during prophage induction in Escherichia coli and found that frequencies of spontaneous and UV induced illegitimate recombination are enhanced by coexpression of RecE and RecT in the wild type, but the enhanced recombination was reduced by recJ,  **recO** , or  **recR**  mutation.  Ref: 12169595 J Bacteriol, 2002
- The newly formed filaments contain an amount of RecA protein and exhibit an ATP hydrolysis rate comparable to that observed when the  **RecO**  and  **RecR**  proteins are included in the initial reaction mixture.  Ref: 9048946 J Mol Biol, 1997
- Bacteriophage lambda can recombine in recBC sbcB sbcC mutant cells by using its own gene orf, the Escherichia coli  **recO** ,  **recR** , and recF genes, or both.  Ref: 7961426 J Bacteriol, 1994
- RecA protein filaments end dependent dissociation from ssDNA and stabilization by  **RecO**  and  **RecR**  proteins.  Ref: 9048946 J Mol Biol, 1997
- Gap repair genes recF,  **recO** , and  **recR**  were not required.  Ref: 10224240 Genetics, 1999
- Cosuppression of recF,  **recR**  and  **recO**  mutations by mutant recA alleles in Escherichia coli cells.  Ref: 7687008 Mutat Res, 1993
- However, lexA Def  **recO**  or lexA Def  **recR**  strains, although unable to facilitate PriA pol II dependent restart, were able to perform pol V dependent readthrough.  Ref: 11929519 Mol Microbiol, 2002
- Several lines of evidence suggest that the recF,  **recO** , and  **recR**  genes function at the same step of recombination and postreplication repair.  Ref: 9537373 J Bacteriol, 1998
- We discuss these results to explain how the RecF  **RecO**   **RecR**  complex functions as an anti Ssb factor.  Ref: 8962075 Proc Natl Acad Sci U S A, 1996
- The  **recO**  mutation did not affect the sensitivity of recF,  **recR**  or recL cells to DNA damaging agents, increased the sensitivity of recU and addAB cells and abolished the DNA repair capacity of recH cells.  Ref: 10323239 Mol Gen Genet, 1999
- Expression of the Escherichia coli mutA mutator phenotype requires recA, recB, recC, ruvA, and ruvC gene, but not recD, recF,  **recO** , or  **recR**  genes.  Ref: 10671469 J Bacteriol, 2000
- The suppression of recF,  **recR**  and  **recO**  mutations by other recA alleles was more pronounced, but none of them could exert full suppression.  Ref: 7687008 Mutat Res, 1993
- With the exception of recA718, all the mutant recA alleles examined were able to suppress the UV radiation sensitivity caused by recF,  **recR**  or  **recO**  mutations, but not by the recB mutation.  Ref: 7687008 Mutat Res, 1993
- The ability of recA718, recA720, recA730, recA750 and two known recA Srf alleles recA2020 and recA441 to act as suppressors of recF,  **recR**  and  **recO**  mutations was examined by studying their UV radiation sensitivity in uvrA cells of Escherichia coli.  Ref: 7687008 Mutat Res, 1993
- In this work, we report that null mutations in recF,  **recO** , or  **recR**  greatly reduce UV radiation mutagenesis UVM in an assay for reversion from a Trp trpE65 to a Trp phenotypes.  Ref: 9537373 J Bacteriol, 1998
- The Escherichia coli RecF,  **RecO**  and  **RecR**  pro teins have previously been implicated in bacterial recombinational DNA repair at DNA gaps.  Ref: 11743007 EMBO J, 2001
- Some proteins, most prominently RecF,  **RecO** , and  **RecR** , have no role in the general formulation.  Ref: 8080625 Crit Rev Microbiol, 1994
- It was found that recF overexpression reduced sulAp expression after DNA damage by mitomycin C or nalidixic acid,  **recO**  and  **recR**  overexpression partially suppressed the reduction of UV induced sulAp expression caused by recF overexpression.  Ref: 7830722 Mol Gen Genet, 1994
- Genetic evidence suggests that the RecF,  **RecO** , and  **RecR**  RecFOR proteins participate in a common step of DNA recombination and repair, yet the biochemical event requiring collaboration of all three proteins is unknown.  Ref: 12769856 Mol Cell, 2003
- We show that normal induction also requires the products of  **recO**  and  **recR** .  Ref: 7862088 Mol Gen Genet, 1995
- Similar to a requirement of recJ in the suppression of recF, the suppression of  **recR**  and  **recO**  mutations by mutant recA alleles also required recJ.  Ref: 7687008 Mutat Res, 1993
- Disassembly is not observed even with ATP when both the  **RecO**  and  **RecR**  proteins are present in the initial reaction mixture.  Ref: 9048946 J Mol Biol, 1997
- Involvement of recF,  **recO** , and  **recR**  genes in UV radiation mutagenesis of Escherichia coli.  Ref: 9537373 J Bacteriol, 1998
- Although the MRF alpha 2 components are apparently not encoded by currently known homologous recombination genes such as recA, recF,  **recO** , and  **recR** , they may fulfill an important function in assembling replisomes on arrested replication forks and products of homologous strand exchange.  Ref: 11459960 Proc Natl Acad Sci U S A, 2001
- Deletion of an open reading frame in the ninR region of lambda results in an additional requirement for  **recO** ,  **recR**  and recF in order to recombine in recBC sbcB sbcC mutant cells.  Ref: 1310087 Genetics, 1992
- Roles of RecJ,  **RecO** , and  **RecR**  in RecET mediated illegitimate recombination in Escherichia coli.  Ref: 12169595 J Bacteriol, 2002
- Addition of recF, recJ, recN,  **recO** , recQ, or  **recR**  mutations had no effect on the survival of ruv recG strains.  Ref: 8132450 J Bacteriol, 1994
- This function, designated orf for  **recO** ,  **recR**  and recF like function, is largely RecF pathway specific.  Ref: 1310087 Genetics, 1992
- iv The recJ, recF,  **recO** , and  **recR**  gene functions are nonessential in this background.  Ref: 8349557 J Bacteriol, 1993
- In the uvrA cells, mutations in recF,  **recR**  or  **recO**  genes produced a major deficiency in the repair of daughter strand gaps.  Ref: 7517004 Mutat Res, 1994
- Using plasmid derivatives that overexpress combinations of the  **recO**  and  **recR**  genes, we tested the hypothesis that high level expression of  **recO**  and  **recR**  recOR in vivo will indirectly suppress the recF mutant phenotypes mentioned above.  Ref: 8206844 J Bacteriol, 1994
- In both uvrA and uvrA recBC sbcBC backgrounds, the UV radiation sensitivity caused by recF, recG,  **recR** ,  **recO** , ruvA, or ruvC mutations was partially suppressed by recA2020.  Ref: 7517004 Mutat Res, 1994
- These observations are incorporated into an expanded model for the functions of RecF,  **RecO** , and  **RecR**  proteins in the early stages of postreplication DNA repair.  Ref: 9363943 Cell, 1997
- Models for the roles of Orf and  **RecO** ,  **RecR**  and RecF in recombination are presented.  Ref: 9335578 Genetics, 1997
- ii this pathway s requires functional recA, recF,  **recO** , and  **recR**  genes, suggesting the mechanism to be daughter strand gap repair.  Ref: 11073901 J Bacteriol, 2000
- The recF,  **recO** , and  **recR**  genes form the recFOR epistasis group for DNA repair.  Ref: 8206844 J Bacteriol, 1994
- The results indicated that RecET mediated illegitimate recombination depends on the functions of RecJ,  **RecO** , and  **RecR** , suggesting that the RecE and RecJ exonucleases play different roles in this recombination pathway.  Ref: 12169595 J Bacteriol, 2002
- Second,  **RecO**  and  **RecR**  are retained in RecA presynaptic filaments and play a role in the subsequent homologous pairing process promoted by RecA.  Ref: 7962001 J Biol Chem, 1994
- **RecR**  protein, which is known to stimulate  **RecO**  protein to facilitate SSB protein displacement by RecA protein, inhibits annealing by  **RecO**  protein, suggesting that  **RecR**  protein may regulate the choice between the DNA strand invasion versus annealing pathways.  Ref: 12438681 Proc Natl Acad Sci U S A, 2002
- The similar phenotypes conferred by recF,  **recR** , and  **recO**  mutations and the observations that a number of mutant recA alleles can cosuppress these three mutations suggest that the recF,  **recR**  and  **recO**  gene products may function together as a complex.  Ref: 7687008 Mutat Res, 1993
- To elucidate the mechanism that underlies this process, we examined protein protein interactions between RecA, RecF,  **RecO** ,  **RecR** , and SSB, and characterized the structure and activity of the ssDNA complexes formed with different combinations of these proteins.  Ref: 7962001 J Biol Chem, 1994
- The  **RecO**  and  **RecR**  proteins thereby stabilize RecA filaments even at the 5 ends of ssDNA, a fact which should affect the recombination potential of 5 ends relative to 3 ends.  Ref: 9048946 J Mol Biol, 1997
- This in turn led to the discovery of several genes, including recF,  **recO** , and  **recR** , whose role in recombination would not otherwise have been detected.  Ref: 8080625 Crit Rev Microbiol, 1994
- The defects in restart attributed to mutations in recF,  **recO**  or  **recR**  were suppressed in a recA730 lexA Def strain expressing constitutively activated RecA RecA\*.  Ref: 11929519 Mol Microbiol, 2002
- The Escherichia coli RecF,  **RecO** , and  **RecR**  proteins were analyzed for their effect on RecA mediated pairing of single stranded circular DNA and homologous linear duplex DNA substrates.  Ref: 8483906 Proc Natl Acad Sci U S A, 1993
- iv When RecA presynaptic filaments were formed in the presence of SSB,  **RecO** , and  **RecR** , the protein DNA complexes obtained contained 70% of the amount of RecA required to saturate ssDNA.  Ref: 7962001 J Biol Chem, 1994
- The resulting  **RecO** . **RecR** .SSB.ssDNA complexes were more active in RecA mediated joint molecule formation than were SSB.ssDNA complexes.  Ref: 7962001 J Biol Chem, 1994
- We propose that mutation of recF, recJ,  **recO** , recQ, or  **recR**  redirects recombination to events initiated by the RecBCD enzyme.  Ref: 8132450 J Bacteriol, 1994
- the  **RecO**  and  **RecR**  proteins also play important roles in the recombination.  Ref: 12169595 J Bacteriol, 2002
- To elucidate the role of several key proteins involved in this process, we have analysed the time dependence of replication restart in strains carrying a combination of mutations in lexA, recA, polB pol II, umuDC pol V, priA, dnaC, recF,  **recO**  or  **recR** .  Ref: 11929519 Mol Microbiol, 2002
- previously isolated mutations in B.subtilis recF, recL,  **recO**  and  **recR** , which belong to the same complementation group, all suppress the lethality of a pcrA mutation.  Ref: 12065426 EMBO J, 2002
- Altered SOS induction associated with mutations in recF,  **recO**  and  **recR** .  Ref: 7862088 Mol Gen Genet, 1995
- **recO**  and  **recR**  mutations delay induction of the SOS response in Escherichia coli.  Ref: 7862097 Mol Gen Genet, 1995
- The introduction of additional mutations in recF, recJ,  **recO** , recQ, or  **recR**  is shown to increase the yield of recombinants by 6 to 20 fold via a mechanism that depends on recBC.  Ref: 8132450 J Bacteriol, 1994
- RecF,  **RecO**  and  **RecR** , three of the important proteins of the RecF pathway of recombination, are also needed for repair of DNA damage due to UV irradiation.  Ref: 7862097 Mol Gen Genet, 1995
- In Escherichia coli, the recovery of replication following UV induced DNA damage is dependent upon several proteins in the recF pathway, including RecF,  **RecO** , and  **RecR** .  Ref: 10589843 Mol Gen Genet, 1999
- In addition, we show that  **recO**  and  **recR**  mutations delay induction of beta galactosidase activity expressed from a lexA regulated promoter following exposure of cells to UV, thus further supporting the idea that recF,  **recO**  and  **recR**  gene products are needed for induction of the SOS response.  Ref: 7862097 Mol Gen Genet, 1995
- The protective effect of plasmid pGam18 against radiation was completely abolished by mutations in the most RecF pathway genes recF, recJ,  **recR** ,  **recO** , recQ, recN, and ruvB.  Ref: 10368783 Genetika, 1999
- Phage lambda has an analog of Escherichia coli  **recO** ,  **recR**  and recF genes.  Ref: 1310087 Genetics, 1992
- RecF protein by itself inhibited reactions in mixtures containing RecA and SSB, and this inhibition was overcome by the addition of  **RecO**  and  **RecR** .  Ref: 8483906 Proc Natl Acad Sci U S A, 1993
- Roles for lambda Orf and Escherichia coli  **RecO** ,  **RecR**  and RecF in lambda recombination.  Ref: 9335578 Genetics, 1997
- Resistance of E. coli strains to cisplatin as determined by plating experiments decreased in the following order parent PQ30 strain, polB >  **recO** ,  **recR** , recF > recB.  Ref: 12531023 DNA Repair Amst , 2002
- This function is countered by the RecF  **RecO**  competition for association with the  **RecR**  protein.  Ref: 11743007 EMBO J, 2001
- In this communication, by measuring the in vivo levels of LexA repressor using anti LexA antibodies, we show that  **recO**  and  **recR**  mutant strains are also not proficient in LexA cleavage reactions.  Ref: 7862097 Mol Gen Genet, 1995
- No evidence for repair was obtained for the wild type or for strains with mutations in the following genes mutS, recA, recBC sbcBC, recD, recF, recJ, recN,  **recO** ,  **recR** , recBC sbcBC recF uvrA, recG ruvC, ruvB, lexA3, lexA51, uvrA, nfo xth nth, polA Ts, or pcnB.  Ref: 8320213 J Bacteriol, 1993
- ii In a recBC sbcA background, in which RecE mediated recombination is active, recA, recJ, recQ, recE, recT, recF,  **recO** , and  **recR**  mutations led to their accumulation.  Ref: 12718760 BMC Mol Biol< MedlineTA>BMC Mol Biol, 1993
- When the  **RecO**  and  **RecR**  proteins are added after most of the RecA protein has already dissociated, RecA protein filaments re form after a short lag.  Ref: 9048946 J Mol Biol, 1997
- Based on these results, we propose dual roles for  **RecO**  and  **RecR**  in joint molecule formation.  Ref: 7962001 J Biol Chem, 1994
- Introduction of the defective lexA51 mutation lexA51 Def and or UmuD into recF,  **recO** , and  **recR**  mutants failed to restore normal UVM in the mutants.  Ref: 9537373 J Bacteriol, 1998
- dnaC suppressors of priA overcome this inviability, especially when RecF,  **RecO**  or  **RecR**  is inactivated, indicating that RdgC avoids or counters a toxic effect of these proteins.  Ref: 12554673 EMBO J, 2003
- This inhibitory effect was overcome by the addition of  **RecO**  and  **RecR**  or RecF,  **RecO** , and  **RecR** .  Ref: 8483906 Proc Natl Acad Sci U S A, 1993
- However,  **RecO**  and  **RecR**  can act together as accessory factors for RecA to overcome this inhibition by SSB Umezu, K.  Ref: 7962001 J Biol Chem, 1994
- Taken together, these results further support the notion that the recF,  **recR**  and  **recO**  gene products abbreviated as RecFOR function at the same step in recombination repair, possible as a complex.  Ref: 7517004 Mutat Res, 1994
- iii RecA can nucleate on the  **RecO** . **RecR** .SSB.ssDNA complexes more efficiently than on SSB.ssDNA complexes.  Ref: 7962001 J Biol Chem, 1994
- Strains carrying  **recR**  insertions combined with mutations in uvrA and other rec genes revealed that the gene is involved in a recombinational process of DNA repair that relies also on recF and  **recO** , and possibly recJ, but which is independent of recB, recC and recD.  Ref: 2664459 Mol Gen Genet, 1989
- Expression of a gene s from the nin region of phage lambda partially complemented both the viability and recombination defects of the recF,  **recO** , and  **recR**  mutants and the recombination defect of ruvC but not of ruvAB or recQ mutants.  Ref: 10735883 J Bacteriol, 2000
- Different DNA damaging agents and amounts of  **RecO**  and  **RecR**  were tested for their effects on this phenotype.  Ref: 7830722 Mol Gen Genet, 1994
- Mutations affecting the RecF pathway of recombination recF, recG, recJ, recN,  **recO** , recQ,  **recR** , ruvA, ruvC were systematically introduced into two sets of strains a uvrA and uvrA recA2020, b uvrA recBC sbcBC and uvrA recBC sbcBC recA2020.  Ref: 7517004 Mutat Res, 1994
- The phage lambda orf gene encodes a trans acting factor that suppresses Escherichia coli  **recO** ,  **recR** , and recF mutations for recombination of lambda but not of E. coli.  Ref: 7961426 J Bacteriol, 1994
- Mutation of recF, recJ,  **recO** , recQ, or  **recR**  improves Hfr recombination in resolvase deficient ruv recG strains of Escherichia coli.  Ref: 8132450 J Bacteriol, 1994
- Finally, examples are shown for in vitro reactions involving DNA, RecA,  **RecO** , RecF,  **RecR** , and SSB that lead to a further understanding of recombinational repair.  Ref: 11098470 Mol Biotechnol, 2000
- A  **recO**  deficient strain did not exhibit dramatic reduction in efficiency of IRR, implying that RecF and  **RecR**  proteins maintain function during this mechanism in the absence of functional  **RecO** .  Ref: 8921869 Gene, 1996
- Furthermore, we find that  **RecO**  and  **RecR**  significantly stimulate this recombination directed DNA replication reaction,.  Ref: 12667462 Mol Cell, 2003
- However, this clone does not complement a  **recO**  mutation for conjugational recombination or  **recO** ,  **recR** , or recF mutations for repair of UV induced DNA damage.  Ref: 7961426 J Bacteriol, 1994
- The recF,  **recO** , and  **recR**  genes were originally identified as those affecting the RecF pathway of recombination in Escherichia coli cells.  Ref: 9537373 J Bacteriol, 1998
- The products of the recF,  **recO** , and  **recR**  genes are thought to interact and assist RecA in the utilization of single stranded DNA precomplexed with single stranded DNA binding protein Ssb during synapsis.  Ref: 8962075 Proc Natl Acad Sci U S A, 1996
- The stimulatory effect of RecF,  **RecO** , and  **RecR**  was not seen without SSB or when SSB was added after RecA.  Ref: 8483906 Proc Natl Acad Sci U S A, 1993
- We compared the recombination proficiencies of recB1080,  **recO** ,  **recR** , and recF single mutants and recB1080  **recO** , recB1080  **recR** , and recB1080 recF double mutants.  Ref: 12618388 Genetics, 2003
- This pathway operating on conjugational recombination requires the recA, recF, recJ, recN,  **recO** , recQ,  **recR** , ruvA, ruvB and ruvC genes.  Ref: 1310087 Genetics, 1992
- These data suggest that  **RecO**  and  **RecR** , and possibly RecF, help RecA overcome inhibition by SSB and utilize SSB single stranded DNA complexes as substrates.  Ref: 8483906 Proc Natl Acad Sci U S A, 1993
- Similarly, recF,  **recO**  or  **recR**  mutations suppress the lethality of the Escherichia coli rep uvrD double mutant.  Ref: 12065426 EMBO J, 2002
- In a wild type, lexA background, mutations in recF,  **recO**  or  **recR**  block both pathways.  Ref: 11929519 Mol Microbiol, 2002
- Since recF is epistatic with  **recO**  and  **recR**  recOR for UV resistance, recOR mutations were also tested with priA2 kan.  Ref: 8820655 Mol Microbiol, 1996
- Bacteriophage lambda lacking its Red recombination functions requires either its own gene product, Orf, or the product of Escherichia coli s  **recO** ,  **recR**  and recF genes RecORF for efficient recombination in recBC sbcB sbcC mutant cells the RecF pathway.  Ref: 9335578 Genetics, 1997
- Induction was delayed to a similar degree by mutation in recF,  **recO**  or  **recR** .  Ref: 7862088 Mol Gen Genet, 1995
- An enhanced function of the  **RecO**  and  **RecR**  proteins can thus be seen in vitro at the 5 ends of linear ssDNA that is not as evident in DNA gaps.  Ref: 11743007 EMBO J, 2001
- In this genetic background, mutations in recF,  **recO** , and  **recR**  had large effects on both cell viability and on recombination.  Ref: 10735883 J Bacteriol, 2000
- Using immunoprecipitation, size exclusion chromatography, and Ssb protein affinity chromatography in the absence of any nucleotide cofactors, we have obtained the following results i RecF interacts with  **RecO** , ii RecF interacts with  **RecR**  in the presence of  **RecO**  to form a complex consisting of RecF,  **RecO** , and  **RecR**  RecF  **RecO**   **RecR** .  Ref: 8962075 Proc Natl Acad Sci U S A, 1996
